# Corporate Social Responsibility and Employee Engagement: Enabling Employees to Employ More of Their Whole Selves at Work

**DOI:** 10.3389/fpsyg.2016.00796

**Published:** 2016-05-31

**Authors:** Ante Glavas

**Affiliations:** Department of Strategy, Sustainability, and Entrepreneurship, Kedge Business SchoolMarseille, France

**Keywords:** corporate social responsibility, engagement, organizational psychology, meaningfulness, perceived organizational support, sustainability

## Abstract

Research at the individual level of corporate social responsibility (CSR) has been growing rapidly. Yet we still lack a more complete understanding of why and how individuals (i.e., employees) are affected by CSR. This study contributes to that gap by exploring the relationship between CSR and employee engagement. Moreover, in order to address the problem of low levels of employee engagement in the workplace, CSR is proposed and tested as a pathway for engaging a significant part of the workforce. Building on engagement theory, a model is tested in which CSR enables employees to bring more of their whole selves to work, which results in employees being more engaged. Data from 15,184 employees in a large professional service firm in the USA was analyzed using structural equation modeling. Results show that authenticity (i.e., being able to show one’s whole self at work) positively and significantly mediates the relationship between CSR and employee engagement. However, the other mediator tested in this study, perceived organizational support (POS; i.e., direct benefits to the employee), did not significantly mediate the relationship. In addition, results of moderated mediation suggest that when CSR is extra-role (i.e., not embedded in one’s job design such as volunteering), it weakens the relationship between CSR and employee engagement. Moreover, *post hoc* analyses show that even when POS is controlled for, authenticity has an impact above and beyond POS on employee engagement. These results extend prior CSR literature which has often been top–down and has focused on how employees will be positively affected by what the organization can give them (e.g., POS). Rather, a bottom–up approach might reveal that the more that employees can give of their whole selves, the more engaged they might be at work.

## Introduction

With studies such as that of Gallup (2013) showing that only 13% of employees are engaged worldwide, engagement is among the lowest it has ever been. On the one hand, employee engagement is a major concern for organizations—just in the USA alone, it is estimated the USA economy loses an estimated 450 to 550 billion USD annually due to decreased productivity from disengaged employees (Gallup, 2013). On the other hand, the lack of employee engagement is also a broader societal issue in that employees are spending more and more time at work, yet if work is not meaningful, it can negatively affect employee well-being (Hulin, 2014). For example, a study by Diener and colleagues (Time, 2005) found that work is not even among the top eight sources of satisfaction in life—a key dimension of subjective well-being.

In parallel, there is a counter-trend emerging in a portion of the workforce in that employees are increasingly engaged at work due to corporate social responsibility (CSR). For example, at Walmart, a company widely criticized for its work conditions, CSR became the main source of employee engagement (Glavas, 2012). One of the initiatives was a Personal Sustainability Plan in which each employee crafted a minimum of one major change they would undertake in order to make their life and work more sustainable—in the end, over 500,000 employees voluntarily participated in CSR initiatives, which also resulted in 35,000 new business solutions that benefitted both the planet and the company (Saatchi and Saatchi S, 2014)^1^. Therefore, scholars have recently begun exploring the CSR–engagement relationship, with studies finding a positive and significant relationship between CSR and employee engagement (e.g., Glavas and Piderit, 2009; Caligiuri et al., 2013). Yet, little is known about why, how, and when employees are engaged by CSR (Glavas, 2016).

Therefore, a theoretical model is tested in this study that is built on engagement theory, which puts forward that the more an individual can show of their whole selves at work, the more they will be engaged (Kahn, 1990; Rich et al., 2010). Two critical engagement factors are tested in this study, which are perceived organizational support (POS) and the ability to be oneself (i.e., authenticity). Prior literature has often focused on employees benefitting from CSR due to the support they will receive (i.e., POS), because it is proposed that companies higher in CSR will be fairer companies and thus treat their employees more fairly (Cropanzano and Rupp, 2008). This study empirically tests the proposed CSR–POS relationship and goes one step further to explore whether employee perceptions of CSR enable them to live out more of their whole selves (i.e., authenticity) at work. In addition, moderated mediation was explored—specifically, whether the relationship between authenticity and engagement is moderated by extra-role involvement in CSR (i.e., volunteering). Although extra-role involvement in CSR might positively affect employees, perhaps too much extra-role involvement in CSR is not a good thing and might be perceived as taking away time from work. To clarify because volunteering—which is used to measure extra-role involvement in this study—has many different forms, for purposes of this study, volunteering is defined as a corporate-sponsored activity of employee involvement in the community and these activities can be initiated by either the employer or employee (Pajo and Lee, 2011).

This study makes the following contributions. It is the first study, based on my review of the literature, to explore the underlying mechanisms (i.e., mediators) between CSR and employee engagement. Second, by unpacking the relationship between CSR and engagement, both positive and negative effects are uncovered. Results suggest that when CSR is embedded, it will more positively affect employees. Third, this is also the first study, to my knowledge, to empirically explore the relationship between CSR and authenticity, finding that CSR enables employees to show more of their whole selves at work. Finally, this study answers the call of Aguinis and Glavas (2012) for more micro-level research on CSR as well as models that include multiple mediators.

## Prior Research on CSR and Employee Engagement

Because the extant CSR literature is broad and diverse, which can lead to confusion regarding the definition of CSR (Peloza, 2009), I define CSR upfront. Based on the definition of Aguinis (2011) and adopted by others (e.g., Rupp, 2011; Rupp et al., 2011; Bauman and Skitka, 2012; El Akremi et al., 2015) CSR is defined as: “context-specific organizational actions and policies that take into account stakeholders’ expectations and the triple bottom line of economic, social, and environmental performance” (Aguinis, 2011, p. 855). CSR is also relevant for a study on engaging the whole self because it is tied to one’s self-concept—as Korschun et al. (2014, p. 24) explain, CSR “reflects a core belief rather than an attitude about a particular social issue.”

Research on CSR and employee engagement is relatively nascent, but there are a few studies that establish that there is a positive relationship between CSR and employee engagement. Glavas and Piderit (2009) found that the effect on employee engagement resulting from positive employee perceptions of CSR was strengthened by importance of CSR to the employee. Caligiuri et al. (2013) also found a positive relationship between CSR and employee engagement; moreover, the authors found a three-way interaction of project meaningfulness, social support, and availability of resources on employee engagement. Glavas (2012) proposed that a reason for the positive relationship between CSR and engagement is that employees find greater meaningfulness and values congruence at work. Specifically, CSR allows for companies to go beyond formal values statements which tend to be words on paper to actually living out these values. This in turn sends signals to employees about the values of the company, which is in line with research that has found a positive relationship between CSR and anticipated values congruence for prospective employees (e.g., Jones et al., 2014). Moreover, CSR can also be a pathway for finding greater meaningfulness at work—in a review of the meaningfulness literature, Rosso et al. (2010) proposed CSR as a pathway through which employees can find meaning because they feel that they are contributing to the greater good. Moreover, Grant et al. (2008) found that the contribution to the greater good makes an employee feel good about themselves, thus improving their own self-concept resulting in greater organizational identification.

Although, there are only a few studies that explore the relationship between CSR and employee engagement, there are studies on related constructs that provide further evidence that there might be a relationship between CSR and engagement. In a study which built a nomological net of employee engagement, job satisfaction and intrinsic motivation were two constructs found to be distinct but related to engagement. Prior CSR research has found a positive relationship between CSR and job satisfaction (e.g., Valentine and Fleischman, 2008; Glavas and Kelley, 2014). Other studies have found a positive relationship between CSR and intrinsic motivation (e.g., Grant, 2008).

In summary, the extant CSR research suggests that there is a relationship between CSR and employee engagement. However, in my review of the literature, I did not find any studies that have explored mediators of the relationship between CSR and employee engagement. In other words, we know that employees can be more engaged due to CSR, but we do not understand the underlying mechanisms.

## Underlying Mechanisms that Explain Why CSR Leads to Employee Engagement

**FIGURE 1:**
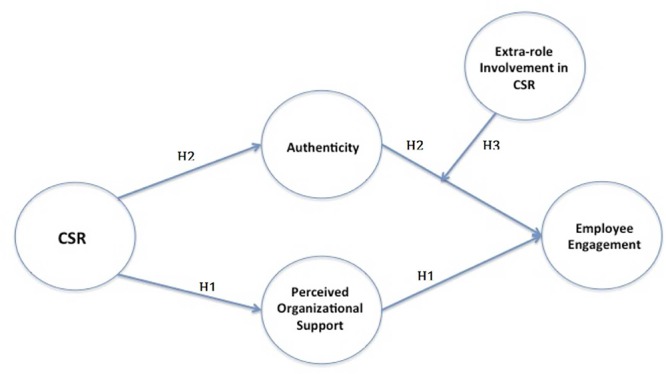
**Multiple mediator and moderated mediation of the relationship between CSR and engagement.** Direct effect of CSR on DVs was also modeled.

Because the focus of this study is on the underlying mechanisms of why employees are engaged, the underlying theory guiding the conceptual framework (see **Figure 1**) builds on engagement theory (e.g., Kahn, 1990; May et al., 2004; Rich et al., 2010). In a review of engagement theory, Saks and Gruman (2014) outlined the different approaches to engagement of which they concluded that theory put forward by Kahn (1990) and later adopted by others (e.g., May et al., 2004; Rich et al., 2010) is the most comprehensive in terms of explaining the underlying psychological mechanisms of engagement. In brief, Kahn’s (1990) approach to engagement is built on theories of the whole self and is based on three underlying mechanisms that influence engagement. The first is related to the content of the work in that employees are more engaged when they are able to do work that is true to themselves, which is referred to as authenticity in this study. Kahn (1990) referred to this content as work that is aligned with what is meaningful to a person. Rich et al. (2010) had a similar reasoning but focused more on values congruence finding that employees are more engaged when they feel that their personal values align with those of the organization. Second, the conditions of work are a key factor in that engagement is influenced by psychological safety, which represents the conditions that enable an employee to show up whole at work (Kahn, 1990). Rich et al. (2010) put forward that POS is the key factor that provides psychological safety. The third is related to traits of the individual which is psychological availability, which is more closely related to self-efficacy and whether an employee has the ability to carry out aspects of their whole selves at work (Rich et al., 2010). Of these three potential underlying mechanisms, the one not related to CSR is psychological availability because it is personal and not influenced by the organization—Rich et al. (2010) measured psychological availability as one’s core self-evaluation (see Judge et al., 2003), which is a stable personality trait. On the other hand, organizations can influence the content (e.g., meaningful work aligned with one’s values) and conditions of work (e.g., POS). In the following text, I put forward hypotheses based on how the content (i.e., authenticity) and conditions (e.g., POS) explain why and how CSR influences employee engagement.

### Perceived Organizational Support

Psychological safety is defined as the ability to show more of one’s whole self without fear of negative consequences (Kahn, 1990). Rich et al. (2010) put forward that POS is critical for psychological safety—in other words, the more that an organization supports an employee, it provides a safe environment in which the employee can be more engaged. When an employee does not believe that there is POS, employees tend to guard themselves, withdraw, and thus disengage (Kahn, 1990; Rich et al., 2010).

Corporate social responsibility could be an antecedent of POS. Numerous empirical studies have found a positive and significant relationship between CSR and POS (Glavas and Kelley, 2014; Shen and Benson, 2014; Ditlev-Simonsen, 2015). One reason is that in a broader stakeholder view of CSR, both external *and* internal stakeholders are cared for, so CSR will lead to POS. Shen and Benson (2014) found that companies high in CSR will also engage in socially responsible human resource management practices. This is also in line with the view that CSR is good management (Waddock and Graves, 1997) in that socially responsible companies are often well-managed companies who find that it benefits the company to treat all of its stakeholders well, including employees. Another reason why CSR is positively related to POS was offered by Cropanzano and Rupp (2008)—the authors build on theories of third-party justice and social exchange theory to propose that when employees see that others are treated fairly, they will also expect to be treated fairly; therefore, employees have higher perceptions of organizational support.

Hypothesis 1. Perceived organizational support mediates the positive relationship between employee perceptions of the organization’s CSR and employee engagement.

### Authenticity

In addition to POS, engagement theory puts forward that employees are more engaged when they perceive congruence with an organization’s values and purpose because they feel as if they are bringing more of their whole selves to work (Kahn, 1990; Rich et al., 2010). In other words, many aspects of the whole self have been lived outside of work (e.g., with family, community, spiritual practices), but the more that work can allow for employees to show their real self, the more engaged they will be (Kahn, 1990; Rich et al., 2010). Turner (1976) defines authenticity as being able to show one’s real self. In other words, authenticity is an antecedent to engagement.

Corporate social responsibility could be an antecedent of authenticity. Korschun et al. (2014) found that the positive effects of CSR are strengthened for employees to whom CSR is connected to their sense of self. Moreover, an important factor for authenticity is values congruence (Rich et al., 2010). Evans et al. (2011) found that employees with other-regarding values were able to find greater values congruence because CSR enabled them to live out these other-regarding values at work, resulting in higher levels of organizational identification and organizational citizenship behaviors. Jones et al. (2014) found that prospective employees were more attracted to organizations higher in CSR because CSR signaled values that were important to them. Another important factor for authenticity is to be able to carry out work that is personally meaningful (Kahn, 1990). Glavas and Kelley (2014) found that because CSR is about serving a higher purpose, employees will find such work meaningful. This also furthers the work of Grant et al. (2008) who found that prosocial identity will mediate the relationship between CSR and affective organizational commitment. The authors put forward that for those employees for whom doing good onto others is important for their self-concept, CSR will be a way through which employees live out more of their whole selves at work.

Hypothesis 2. Authenticity mediates the positive relationship between employee perceptions of the organization’s CSR and employee engagement.

### Extra-Role Involvement

In addition to POS and authenticity as potential mediators of the CSR—employee engagement relationship, extra-role behaviors such as volunteering could moderate this relationship. For example, Caligiuri et al. (2013) found that volunteering led to increased engagement and was strengthened by availability of resources and project meaningfulness—employees were able to live out more of their real selves through volunteering projects. Jones (2010) found that volunteering increased organizational identification mediated by organizational pride (i.e., seeing how one’s work benefited the community made an employee feel proud of their organization); in turn employee organizational citizenship behaviors were increased. Muthuri et al. (2009) found that volunteering positively influenced employees due to improved social capital.

Although these studies suggest that there is a positive impact of volunteering on employees, what has not been studied as extensively is if too much extra-role behavior can have a negative impact. Aguinis and Glavas (2013) proposed that employees are more positively impacted by involvement in CSR when it is embedded into one’s job; however, when CSR is peripheral (i.e., extra-role) it can have a negative impact on employees. Employees might perceive CSR as being disingenuous. Moreover, extra-role CSR might put pressure on employees who already have high job demands. For example, Grant (2012) found that if there is too much pressure for volunteering that it can have a negative impact on employees. This is what Pierce and Aguinis (2013) would describe as too much of a good thing effect.

Hypothesis 3. Extra-role involvement (i.e., volunteering) in CSR will moderate the positive relationship between authenticity and employee engagement, such that the relationship will be weakened by increased extra-role involvement in CSR.

## Materials and Methods

### Ethics Statement

The study design and processes used to protect the interests and rights of the human subjects involved in this study was deemed as exempt by the Institutional Review Board at The University of Notre Dame.

### Setting and Sample

Participants were 15,184 employees from a large professional services firm in the USA. Survey responses were collected as part of an annual workplace survey. The response rate was 73.3%. Due to legal restrictions by the company, I was not given access to individual demographic data. However, the company did disclose the overall demographics of the sample and 48.6% of participants were female and the mean tenure was 6.5 years, which was representative of general company demographics.

### Procedure

Because the primary goal was to analyze the data at the individual level, intraclass correlations (ICCs) were calculated in order to rule out office-level effects. ICC values ranged from 0.007 to 0.032. Despite the low ranges, I still included office as a control variable.

In addition, due to high correlations between variables, collinearity statistics were analyzed. The highest variance inflation factor (VIF) was 3.20, which is well below the recommended cutoff of 10 (Ryan, 1997).

To test the hypotheses, structural equation modeling was employed with Mplus Version 7 (Muthén and Muthén, 2012). The approach to mediation and moderated mediation analysis was done based on guidelines by Hayes (2013) and Stride (2015). Bootstrapping with 1000 replications was used to obtain standard errors, estimates, and bias-corrected 95% confidence intervals according to procedures recommended by Preacher and Hayes (2008). The direct effect of CSR on the dependent variable (i.e., engagement) was also modeled.

### Measures

The measures were developed as part of the company’s annual workplace survey and are adapted from the Great Place to Work Survey which has been used in prior research (see Fulmer et al., 2003). Each item, except extra-role involvement in CSR, was measured on a scale of 1 (rarely) to 5 (almost always). Internal consistency reliabilities (i.e., Cronbach’s alphas) for each scale are presented in **Table 1**.

**Table 1:** Descriptive statistics, reliability estimates, and study variable intercorrelations.

Variable	*M*	*SD*	1	2	3	4	5	6	7
(1) Corporate social responsibility	3.99	0.73	(0.81)						
(2) Perceived organizational support	3.84	0.77	0.68^∗∗∗^	(0.79)					
(3) Authenticity	3.96	0.77	0.76^∗∗∗^	0.76^∗∗∗^	(0.81)				
(4) Engagement	3.96	0.86	0.68^∗∗∗^	0.66^∗∗∗^	0.74^∗∗∗^	(0.90)			
(5) Pay satisfaction	3.39	0.92	0.57^∗∗∗^	0.63^∗∗∗^	0.60^∗∗∗^	0.64^∗∗∗^	(0.82)		
(6) Satisfaction with recognition	3.63	0.96	0.62^∗∗∗^	0.65^∗∗∗^	0.69^∗∗∗^	0.64*^∗∗∗^*	0.57^∗∗∗^	(0.77)	
(7) Satisfaction with leadership	3.93	0.70	0.73^∗∗∗^	0.70^∗∗∗^	0.78^∗∗∗^	0.77^∗∗∗^	0.61^∗∗∗^	0.67^∗∗∗^	(0.92)

*Scales are from 1 to 5. Coefficient (α) reliabilities are shown in the diagonal. ^∗^p < 0.05, ^∗∗^p < 0.01, ^∗∗∗^p < 0.001*.

#### Independent Variable

The independent variable, CSR, was measured with five items such as “I believe [my company] makes a positive contribution to the communities in which it operates,” and “[My company] demonstrates a clear commitment to its environmental initiatives.”

#### Dependent Variable

The dependent variable, employee engagement, was measured with four items. This scale has previously been used and validated (Block et al., in press) with the scale found to map onto the emotional dimension of the employee engagement scale of Rich et al. (2010). Items were such as “Overall, I would say that this is a great place to work,” and “I rarely think about looking for a new job with another organization.”

#### Mediators

Authenticity was measured with four items such as “I can be myself at work,” and “There is an emphasis on integrity here.” POS was measured with four items such as “I get fair consideration for the best engagements or assignments,” and “If I feel that I am treated unfairly, I am comfortable going to management to address my concerns.”

#### Moderator

Extra-role involvement in CSR was measured with the following item: “Please indicate the approximate number of hours you spend annually participating in firm-sponsored or personal community service/philanthropic activities.” To clarify the terminology that the sample firm uses, firm-sponsored activities are a few strategic initiatives that are encouraged throughout the firm. Personal activities are those that are employee initiated but still conducted officially on behalf of the firm.

#### Control Variables

I also controlled for other key variables that might influence employee attitudes such as satisfaction with leadership, pay satisfaction, and satisfaction with recognition. Satisfaction with leadership was measured with nine items such as “Management does an effective job of operating the business.” Pay satisfaction was measured with three items such as “I am paid fairly for the work I do.” Satisfaction with recognition was measured with two items such as “Management recognizes and shows appreciation for quality work and extra effort.”

## Results

### Descriptive Statistics

Means, standard deviations, reliabilities, and intercorrelations among the variables are presented in **Table 1**.

### Hypothesis Testing

The overall model showed acceptable fit. The root-mean-square error of approximation (RMSEA) was 0.072 with 90% confidence intervals of 0.071 and 0.073. The standardized root mean square residual (SRMR) for the model was 0.041. The comparative fit index (CFI) for the model was 0.89 and the Tucker and Lewis Index (TLI) was 0.88.

**Table 2** reports indirect effects with unstandardized estimates, corresponding standard errors, and corresponding bias-corrected 95% confidence intervals. Hypothesis 1 was not supported. In other words, POS did not significantly mediate the relationship between CSR and employee engagement. Hypothesis 2 was supported. Authenticity positively and significantly mediated the relationship between CSR and employee engagement.

**Table 2:** Results of mediation tests predicting employee engagement: indirect effects of CSR through two mediators (perceived organizational support and authenticity).

			BC 95% CI	
Indirect and direct effects	Estimate	*SE*	Lower	Upper
**Indirect effects**				
CSR → Perceived Organizational Support → Engagement (H1)	-0.642	0.108	-0.882	-0.465
CSR → Authenticity → Engagement (H2)	**1.719**	0.439	1.090	2.792
**Direct effects**				
CSR → Engagement	-0.341	0.388	-1.355	0.162
CSR → Perceived Organizational Support	**1.144**	0.045	1.058	1.233
CSR → Authenticity	**1.612**	0.059	1.499	1.727
Perceived Organizational Support → Engagement	-0.561	0.086	-0.745	-0.410
Authenticity → Engagement	**1.066**	0.289	0.657	1.764

*BC 95% CI refers to the bias-corrected 95% confidence interval; Estimate refers to the effect estimate using 1,000 bootstrap samples; estimates with CIs that do not include zero are statistically significant and bolded; CSR, corporate social responsibility.*

Hypothesis 3 was supported. Extra-role involvement in CSR weakened the relationship between authenticity and employee engagement. To test Hypothesis 3, I used the procedure for moderated mediation proposed by Hayes (2013) and Stride (2015) for Mplus. The interaction term (extra-role involvement in CSR) was significant (β = -0.042, *p* < 0.01) and predictors explained 67.5% of the variance of employee engagement. I operationalized high and low scores as 1 SD above and below the mean score. The estimates, standard errors, and 95% confidence intervals for the conditional indirect effects are presented in **Table 3**.

**Table 3:** Results for test of conditional indirect effects of CSR-engagement through a mediator (authenticity) at specific values of the moderator (extra-role involvement in CSR): mean ± 1 standard deviation.

			95% CI	
Value of extra-role involvement in CSR	Conditional indirect effect	*SE*	Lower	Upper
-1 *SD* (1.53)	**0.082**	0.005	0.073	0.093
*M* (2.69)	**0.080**	0.006	0.068	0.092
+1 *SD* (3.85)	**0.077**	0.008	0.063	0.092

*95% CI refers to the 95% confidence interval; estimate refers to the effect estimate using 1,000 bootstrap samples; estimates with CIs that do not include zero are statistically significant and bolded; CI refers to the bias-corrected 95% confidence interval.*

### *Post hoc* Analyses

#### Common Method Bias

To control for common method bias, I first conducted a Harman’s single-factor test (Podsakoff et al., 2003) with the one factor accounting for less than 50% of the variance. Because of the critique of the Harman’s test, I further conducted a *post hoc* analysis using the marker variable technique (Lindell and Whitney, 2001; Podsakoff et al., 2003), in which I partialed out the effect of a theoretically unrelated marker variable (market development). As expected, the fit was a bit worse, but still acceptable, compared to the baseline model (RMSEA = 0.084, 90% confidence intervals of 0.084 and 0.085; SRMR = 0.133; CFI = 0.85; TLI = 0.83), and all the path estimates from the previous model that were significant (i.e., Hypotheses 2 and 3), remained significant and in the same direction.

#### Controlling for POS

Because Glavas and Kelley (2014) found that CSR affects work meaningfulness above and beyond POS, I also tested whether there is an indirect effect of CSR on engagement through authenticity that goes above and beyond the influence of POS. I tested the baseline model but instead of POS being a mediator, I controlled for POS. Authenticity still mediated the relationship between CSR and employee engagement (β = 1.443, *p* < 0.001).

#### Reverse Causality

Because it is possible that engaged employees might have more positive perceptions of the organization (e.g., CSR, authenticity, POS), reverse causality was analyzed. The same baseline model shown in **Figure 1** was tested in reverse with the exception of moderated mediation. None of the indirect paths were significant. The overall model showed similar fit to the baseline model in this study. The RMSEA was 0.073 with 90% confidence intervals of 0.072 and 0.074. The standardized root mean square residual (SRMR) for the model was 0.042. The CFI for the model was 0.89 and the TLI was 0.88. The indirect path from employee engagement to perceived CSR, mediated by POS was insignificant and negative (*b* = -0.012, *p* = 0.104). The indirect path from employee engagement to perceived CSR, mediated by authenticity was also insignificant (*b* = 0.026, *p* = 0.183).

#### Main Effect of CSR and Engagement with No Mediators

Because many studies between CSR and employee outcomes have not included mediators, I tested the relationship using the baseline model (i.e., same controls and analysis) but without mediators. The relationship between CSR and engagement was found to be positive and significant (β = 0.837, *p* < 0.001). This is counter to the findings from the baseline model in this study (i.e., full model with mediators) in which the direct effect between CSR and engagement is not significant.

## Discussion

In this study, I found a positive and significant relationship between employee perceptions of CSR and employee engagement, which was mediated by authenticity. The other mediator, POS, did not significantly mediate the CSR—engagement relationship and the relationship was actually negative. Moreover, when POS was controlled for in the *post hoc* analyses, authenticity had an effect above and beyond that of POS on employee engagement. These findings suggest that perceived CSR has the strongest impact on employees when it allows for them to show their whole selves at work (i.e., authenticity). Moreover, when employees perceive that POS is related to CSR, it might have a negative impact. In addition, extra-role involvement in CSR was found to weaken the effect of authenticity on employee engagement. These results suggest that even if employees are positively affected by CSR, they prefer that CSR does not entail work above and beyond their own job.

### Theoretical Implications

Based on my review of the literature, this is the first study to explore underlying mechanisms between employee perceptions of CSR and engagement. Moreover, this is the first study to my knowledge that directly tested the relationship between CSR and authenticity—defined as the ability to bring one’s whole self to work. As predicted by prior engagement theory (e.g., Kahn, 1990; Rich et al., 2010), authenticity did mediate the relationship between CSR and engagement. But contrary to engagement theory (e.g., Kahn, 1990; Rich et al., 2010) and prior CSR research on POS (e.g., Shen and Benson, 2014; Ditlev-Simonsen, 2015), POS was not found to be significantly related to engagement. This has important implications for CSR and organizational psychology because it goes beyond a top–down model in which the direct benefits of CSR to the employee (e.g., POS) predict how the employee will be affected. Instead, a bottom–up model in which employees can give more of their whole selves might have a stronger effect on employees. These findings also highlight the importance of going beyond studying the influence of external factors (e.g., POS) to studying how intra-individual factors (e.g., authenticity) influence how employees are affected by CSR.

Moreover, a bottom–up approach to CSR is one in which CSR is embedded in one’s job. As the results of this study suggest, when CSR is extra-role it can have negative effects on employees. These findings have implications for CSR theory which has primarily built models based on the strategy and policies of an organization without taking into consideration if and how CSR is embedded into the jobs of employees (Aguinis and Glavas, 2013). By exploring the degree of CSR embeddedness, both positive and negative effects of CSR on employees can be uncovered.

Finally, this study contributes to gaps identified in a review of the CSR literature by Aguinis and Glavas (2012) who proposed that a more complete picture of CSR should be built in which the individual level of analysis is included. Prior CSR research has mostly been at the macro and institutional levels (Lee, 2008; Wood, 2010). In addition, this study includes multiple mediators, which are rarely studied in CSR at the individual level, but important to explore in order to understand how different mechanisms influence employees (Jones et al., 2014). Third, moderators are analyzed through moderated mediation, which addresses the need for exploring moderators of the CSR–employee outcomes relationship (Rupp et al., 2013). This has important theoretical implications because when effects of CSR on employees are aggregated to the macro level (i.e., without including mediators and moderators at the individual level of analysis), both positive and negative effects on employees are confounded. Perhaps this is why the macro CSR literature has led to inconclusive findings on whether CSR has a positive relationship with organization outcomes (Wood, 2010; Aguinis and Glavas, 2012). By understanding why, how, and when employees are positively and negatively affected by CSR, more complete models of CSR can be built in which the positive effects of CSR can be disentangled.

### Managerial Implications

Mirroring theoretical implications, CSR should be embedded in practice as much as possible. Too often, CSR programs are put together by a department on the periphery of the company that emphasizes extra-role CSR behavior such as volunteering, recycling, and similar initiatives. Rather CSR could be part of one’s job through two possible ways. CSR could be embedded throughout the organization (Aguinis and Glavas, 2013) such that it is part of an organization’s strategy, products, and services. This is rare and at best, often organizations are somewhere on the path toward embedding CSR, but it is a journey that many organizations do embark on (Aguinis and Glavas, 2013). The other path, which can also be in parallel, is bottom up. Employees can embed CSR in their own jobs through models of job crafting (for job crafting and CSR, see Sonenshein et al., 2014).

Second, the findings suggest that CSR should be more individualized and personal. Often companies have a unified strategy for implementing CSR organization-wide. However, if we take the findings of this study, then CSR is something that can really move people at a deep level. CSR can connect to what is most meaningful for a person and to their core values. Because each individual is different, CSR should be individualized. As a result, the firm also benefits as a part of the workforce can be re-energized. If even 13% of the workforce can be re-engaged, that is also a huge economic benefit to organizations. As the Gallup (2013) report found, which was conducted on 230,000 employees in 142 countries, only 13% of the current workforce is engaged. Engaging an additional 13% will double the amount of engaged employees. Moreover, Gallup (2013) calculated that due to population growth and GDP increase, there will be $140 trillion in new customers. Moreover, the authors proposed that those companies that are able to engage their employees more will have a competitive advantage in this new marketplace.

Finally, all this goes without saying that perhaps the stakeholder that “wins” the most is the employee. If CSR is about improving the well-being of others, then enabling employees to find well-being through work, the activity that takes the most time out of many people’s lives, is a CSR achievement in and of itself.

### Future Research and Limitations

The measure of extra-role involvement in CSR conflated both involvement in firm-initiated strategic corporate volunteering initiatives as well as employee-initiated corporate volunteering initiatives. Future research could disentangle these two in order to explore if initiatives proposed by employees might have positive effects due to its discretionary nature. For example, employees might be able to design initiatives that are more aligned to their whole self (e.g., values, perceptions of meaningful work).

In addition, the relationship between CSR and authenticity could be explored in much more depth. Because authenticity is intra-individual by its very nature, intra-individual factors could be explored. For example, it might be interesting to study whether CSR leads to authenticity because it influences values alignment, meaningfulness at work, and/or aligns with an employee’s identity (e.g., prosocial identity)—and for whom. The latter could be studied by exploring the role of individual differences such as other orientation and conscientiousness.

Future research could also explore how the social exchange relationship between employees and the organization changes when authenticity is introduced into the model. Although, this study did not explore social exchange theory, conceptual frameworks that include POS often build on those of social exchange theory (Cropanzano and Mitchell, 2005). However, the starting point is often the organization and what it does to the employee, thus leading to a reciprocal exchange. It might be interesting to explore if this relationship changes when the starting point is the employee and they are enabled to show more of their whole selves at work.

Finally, there are limitations that apply to this study that can be overcome with future research. For example, the cross-sectional design of this study could be addressed with studies such as those that include other ratings, are experimental, and/or longitudinal. Moreover, because this study was on a single firm in the U.S., other studies could be conducted in multiple firms (of varying size), industries, and countries. For example, the findings in this study might differ in more blue collar employee populations. In addition, it would be interesting to compare the findings to data from firms in which CSR is highly embedded. Also, the measures used in this study were ones that were used as part of the organization’s annual survey. Other established measures for the variables in the model could be tested as well, including collecting demographic variables, which were not disclosed due to legal privacy regulations.

## Conclusion

Engagement theory has primarily focused on the relationship between the individual and the organization. CSR theory has primarily focused on the relationship between the organization and society. By combining both, more complete multilevel models of not only CSR, but management in general can be built that are holistic in nature. As a result the individual benefits, the organization benefits, and society benefits.

## Author Contribution

The author confirms being the sole contributor of this work and approved it for publication.

## Conflict of Interest Statement

The author declares that the research was conducted in the absence of any commercial or financial relationships that could be construed as a potential conflict of interest.

## Footnotes

1 http://cdn.corporate.walmart.com/cd/4e/83b19cf8481392311929c7fbf315/r_1363.pdf
